# Temporal Trends in Distal Symmetric Polyneuropathy in Type 2 Diabetes: The Fremantle Diabetes Study

**DOI:** 10.1210/clinem/dgad646

**Published:** 2023-11-01

**Authors:** Wendy A Davis, Emma Hamilton, Timothy M E Davis

**Affiliations:** Medical School, University of Western Australia, Fremantle Hospital, Fremantle, WA 6959, Australia; Australian Centre for Accelerating Diabetes Innovations (ACADI), The University of Melbourne, Melbourne, VIC 3010, Australia; Medical School, University of Western Australia, Fremantle Hospital, Fremantle, WA 6959, Australia; Department of Endocrinology and Diabetes, Fiona Stanley and Fremantle Hospitals Group, Murdoch, WA 6150, Australia; Medical School, University of Western Australia, Fremantle Hospital, Fremantle, WA 6959, Australia; Australian Centre for Accelerating Diabetes Innovations (ACADI), The University of Melbourne, Melbourne, VIC 3010, Australia; Department of Endocrinology and Diabetes, Fiona Stanley and Fremantle Hospitals Group, Murdoch, WA 6150, Australia

**Keywords:** type 2 diabetes, distal symmetric polyneuropathy, temporal trends, community-based, longitudinal study

## Abstract

**Context:**

Macrovascular outcomes in type 2 diabetes have improved over recent decades. There are scant equivalent distal symmetric polyneuropathy (DSPN) data.

**Objective:**

This work aimed to characterize temporal changes in DSPN prevalence and incidence rates (IRs) in community-based Australians.

**Methods:**

An observational study was conducted among an urban population. Participants included individuals with type 2 diabetes from the Fremantle Diabetes Study phases I (FDS1; n = 1296 recruited 1993-1996) and II (FDS2; n = 1509 recruited 2008-2011). Main outcome measures included Michigan Neuropathy Screening Instrument (MNSI) clinical grading.

**Results:**

DSPN prevalence by 8-point MNSI was 30.8% (FDS1) and 58.9% (FDS2; *P* < .001), and by 6-point (excluding foot appearance) and 2-point (biothesiometry alone) MNSI was 37.5% and 35.7% (*P* = .336), and 33.8% and 38.7% (*P* = .011), respectively. Given between-phase changes in appearance assessment, 8-point MNSI data were not analyzed further. In multivariable analysis, FDS2 vs FDS1 participation was associated with 6-point (odds ratio (95% CI) 0.68 (0.56-0.83); *P* < .001) but not 2-point (0.90 (0.74-1.11); *P* = .326) MNSI DSPN prevalence. Four-year DSPN IRs (95% CI) for 6-point MNSI were 13.6 (12.0-15.4) and 17.6 (15.9-19.4)/100 person-years in FDS1 and FDS2, respectively (IR ratio [IRR] 1.31 [1.12-1.55]; *P* < .001), and for 2-point MNSI were 13.9 (12.3-15.8) and 7.4 (16.3-8.6/100 person-years; IRR 0.53 [0.43-0.64]; *P <* .001). FDS2 vs FDS1 independently predicted incident DSPN for 6-point (hazard ratio [95% CI] 1.25 [1.06-1.48]; *P* = .009) and 2-point (0.42 [0.33-0.55]; *P* < .001) MNSI.

**Conclusion:**

DSPN prevalence was lower or equivalent in FDS2 vs FDS1, and its incidence was greater or lower, in multivariable models depending on the MNSI features used.

There has been a recent decline in the burden of chronic macrovascular complications of type 2 diabetes in high-income countries (1-4), including Australia (5, 6), consistent with improved clinical management (7-9). Although these trends may have plateaued and even reversed in younger age groups in the United States (10, 11), available data show that there have been relatively consistent reductions in overall rates of cardiovascular disease (CVD) events and death in many countries (12). The equivalent temporal changes in microvascular complications are, however, less clear. Although there are limited relevant data, there is some evidence that the prevalence of, and progression to, more severe forms of diabetic retinopathy has reduced over the last 3 decades (13). Most studies of nephropathy have used rates of dialysis and transplantation for end-stage kidney disease, which are increasing (12), an observation complicated by increased health system willingness to implement renal replacement therapy in older individuals with comorbidities and perhaps a more severe clinical phenotype in younger patients (10, 14, 15).

In the case of distal symmetric polyneuropathy (DSPN), there are few, if any, valid longitudinal data that allow an assessment of temporal trends (12). As DSPN is a major risk factor for lower extremity amputation (LEA), especially more distal procedures (16), declining LEA rates including minor amputations (16, 17) may be a surrogate for the same trend in DSPN. However, other complications of diabetes are risk factors for LEA, including CVD in the form of peripheral arterial disease and end-stage kidney disease (16, 18), and they have exhibited variable changes in incidence over recent decades (12). Recently published longitudinal data from a Danish tertiary diabetes center suggest that the incidence of DSPN is declining in heavily selected individuals referred with complex type 2 diabetes (19). Given this background, there is a need for longitudinal data in which DSPN has been ascertained using validated measures in a representative community-based cohort.

To address the lack of relevant data, we have used baseline and longitudinal outcome data from the Fremantle Diabetes Study phases I (FDS1) and II (FDS2) to determine whether the incidence of DSPN in Australians with type 2 diabetes has changed in the 15 years between the 2 phases and to examine the independent predictors of DSPN within the pooled FDS type 2 diabetes cohorts including the potential influence of FDS phase.

## Materials and Methods

### Participants and Approvals

The FDS1 is an observational, longitudinal cohort study of known diabetes conducted in a zip code–defined area surrounding the city of Fremantle in Western Australia (20). Participants were recruited between 1993 and 1996, with annual face-face-to face follow-up visits scheduled for 5 years post recruitment. The FDS2 used the same design as FDS1 (20), with recruitment between 2008 and 2011, but with in-person assessments every 2 years up to 6 years post recruitment. Participants in both phases were identified from a variety of sources including hospital, clinic, and primary care patient lists; local media advertising; pharmacies; optometrists; networks of health care professionals including podiatrists; and, in the case of FDS2, third-party mail-outs to registrants of the Australian National Diabetes Services Scheme and the National Diabetes Register (20). Details of recruitment, sample characteristics including classification of diabetes types, and nonrecruited people identified with type 2 diabetes in the catchment area have been published previously (20). The FDS1 protocol was approved by the Fremantle Hospital Human Rights Committee, and the FDS2 protocol by the Human Research Ethics Committee of the Southern Metropolitan Area Health Service. All participants gave written informed consent.

### Baseline and Follow-up Assessments

In both FDS phases, assessment at entry and at each annual (FDS1) or biennial (FDS2) review included a comprehensive questionnaire, a detailed physical examination by trained registered nurses, and fasting biochemical tests performed in a single nationally accredited laboratory (20). In addition to details of all medical conditions, demographic, socioeconomic, and lifestyle data were recorded. Full details of all medications were recorded. A Body Shape Index (ABSI) was calculated as a more robust index of visceral obesity than body mass index (BMI) (21). Complications were identified using standard definitions (22). Renal function was assessed from the estimated glomerular filtration rate (eGFR) (23). Participants were classified as having prevalent coronary heart disease (CHD) if there was a history of myocardial infarction, angina, coronary artery bypass grafting, or angioplasty, and as having prevalent cerebrovascular disease if there was a history of stroke and/or transient ischemic attack. Peripheral arterial disease was defined as an ankle brachial index of 0.90 or less or the presence of a diabetes-related LEA.

### Ascertainment of Distal Symmetric Polyneuropathy in the Fremantle Diabetes Study Phase 1 and Fremantle Diabetes Study Phase 2

DSPN was ascertained using the clinical portion of the Michigan Neuropathy Screening Instrument (MNSI) (24). The MNSI score comprises 4 parameters in each foot, specifically appearance (deformities, dry skin, callus, fissure or infection; grading 0 normal, 1 abnormal), presence of ulceration (0 normal, 1 present), reflexes (0 normal, 0.5 present with reinforcement, 1 absent), and vibration originally assessed through use of a tuning fork (24) but measured in the present study by vibration perception threshold (VPT) determined using biothesiometry at the great toe (0 present [<20 V], 0.5 reduced [20-30 V], 1 absent [>30 V]), giving a total score out of 8.

Although the MNSI has been validated against nerve conduction studies (NCS) (24-26), there are potential issues with its application in specific circumstances, especially grading of foot appearance, which may depend on age, location and, in the present study, timing in relation to the FDS phases. For example, xerosis cutis of the feet affects a substantial majority of nursing home residents (27), while 1 in 6 Australians report callus or corns (28) with at least a doubling of this prevalence in older age groups (29). These are much higher proportions than in the United States (30, 31), likely reflecting the regular use of open footwear by more than 25% of Australian adults (32, 33). A high background rate of MNSI appearance abnormalities in the general population may artifactually increase DSPN ascertainment. Australian government-subsidized primary care initiatives, including availability of podiatry assessments, were introduced between FDS phases (34). Although this may have improved foot health between FDS1 and FDS2, it may also have increased patient and health practitioner awareness of MNSI foot appearance abnormalities (35). The prevalence of foot ulceration in community-based individuals with type 2 diabetes in Australia is less than 2% (36), suggesting that its presence contributes proportionately little to the MNSI compared with other features. Ankle jerks are more difficult to elicit in older individuals in the general population even when standardized procedures are used (37, 38). VPT also increases with age (39), but it has been used as a single modality to assess the presence of DSPN in well-regarded intervention trials (40), compares favorably against NCS (41), and has good interrater reliability (42, 43).

### Statistical Analysis

The computer packages IBM SPSS Statistics 28 (IBM Corporation) and StataSE 15 (StataCorp LP) were used for statistical analysis. Data are presented as proportions, mean ± SD, geometric mean (SD range), or, in the case of variables that did not conform to a normal or log-normal distribution, median and interquartile range. Two-sample comparisons were by Fisher exact test for proportions, *t* test for normally distributed variables, and Mann-Whitney *U* test for other variables. Multiple logistic regression (backward stepwise conditional with entry *P* < .050 and removal *P* ≥ .050) was used to identify independent associates of prevalent DSPN that were considered for model entry based on clinical plausibility and *P* less than .20 in bivariable analyses.

Given that there were coincident biennial face-to-face assessments up to year 4 in FDS1 and FDS2, 4-year incident rates (IRs) for each DSPN category were derived for each FDS phase. Incident rate ratios (IRRs) were then calculated for FDS2 vs FDS1 and incident rate differences (IRDs) were also determined. To determine whether IRs for each DSPN outcome differed significantly by FDS phase, interval-censored Weibull regression (backward stepwise conditional with entry *P* < .050, removal *P* ≥ .050) was used incorporating clinically plausible baseline variables with *P* less than .20 in bivariable analyses. To assess the effect of FDS phase, participation in FDS2 vs FDS1 was then added to each most parsimonious model. Missing values were multiply imputed (×20).

## Results

### Participant Characteristics

In FDS1, 2258 people with diabetes were identified from a population of approximately 120 000, and 1426 (63%) were recruited, of whom 1296 (91%; mean age 64 years, 49% male, median diabetes duration 4.0 years) had clinically defined type 2 diabetes. In FDS2, 4639 people with diabetes were identified from a population of approximately 157 000, and 1668 (36%) recruited, of whom 1509 (90%; mean age 65 years, 52% male, median diabetes duration 8.0 years) had type 2 diabetes.

### Prevalence of Distal Symmetric Polyneuropathy

**Figure 1.**
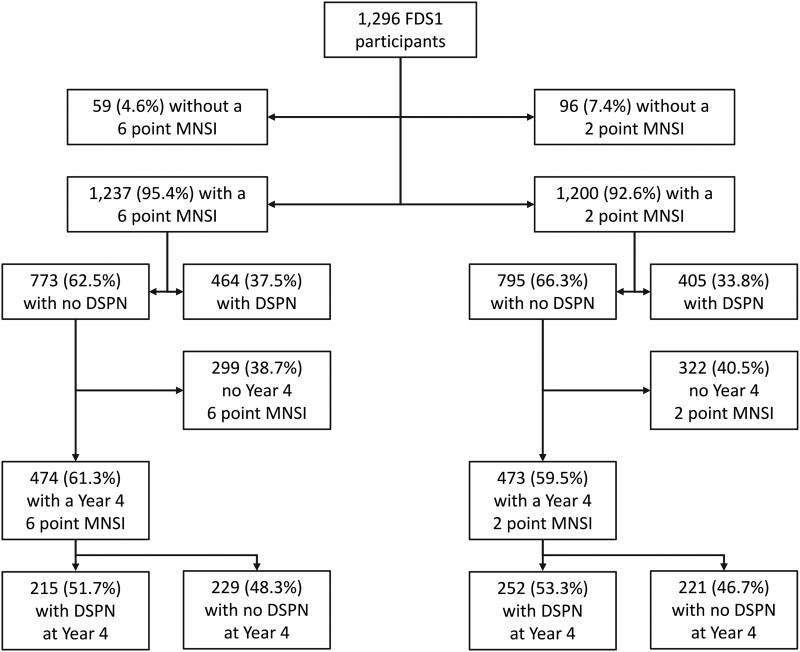
Consort diagram showing disposition of Fremantle Diabetes Study Phase I (FDS1) participants assessed for distal symmetric polyneuropathy (DSPN) by 6- and 2-point Michigan Neuropathy Screening Instrument (MNSI) scores at baseline, year 2, and year 4. If participants had incident DSPN at year 2, it was assumed that this was also present at year 4 if they did not attend for assessment.

**Figure 2.**
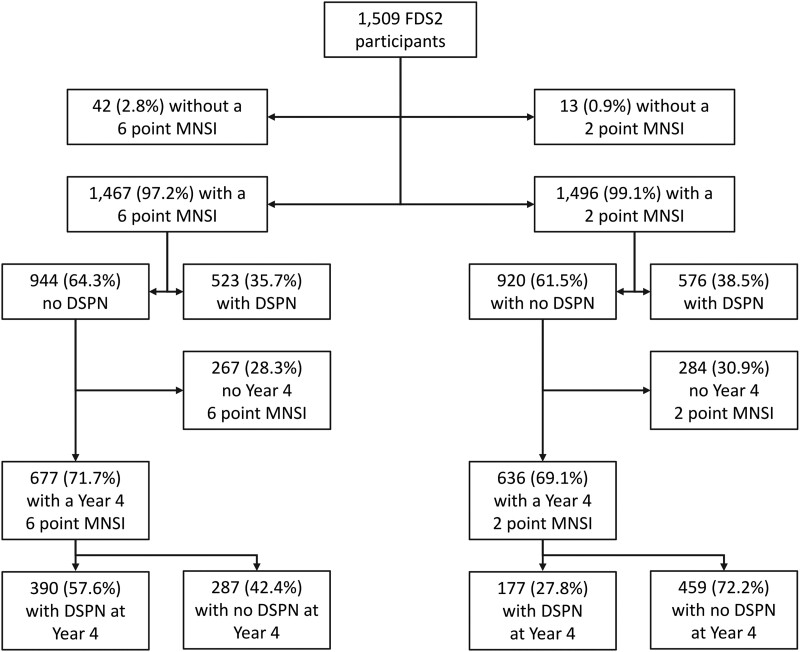
Consort diagram showing disposition of Fremantle Diabetes Study Phase 2 (FDS2) participants assessed for distal symmetric polyneuropathy (DSPN) by 6- and 2-point Michigan Neuropathy Screening Instrument (MNSI) scores at baseline, year 2, and year 4. If participants had incident DSPN at year 2, it was assumed that this was also present at year 4 if they did not attend for assessment.

The prevalence of DSPN using the full 8-point MNSI score was 30.8% in FDS1 and 58.9% in FDS2 (*P* < .001). The respective FDS1 vs FDS2 figures for 6-point MNSI (excluding foot appearance with DSPN present at a score >1.5) and 2-point MNSI (using VPT alone with DSPN present at a score >0.5) were 37.5% and 35.7% (*P* = .336; Fig. 1), and 33.8% and 38.7% (*P* = .011; Fig. 2). These comparisons suggested that the subjective assessment of foot appearance had changed significantly between phases, as discussed earlier, and therefore 6- and 2-point scores were used in subsequent analyses.

The characteristics of pooled participants in FDS1 and FDS2 by prevalent DSPN status assessed by 6-point or 2-point MNSI score are shown in Table 1. There was a range of significant bivariable associations that were shared across the 2 MNSI gradings. Compared to those without DSPN, participants with DSPN were older, more likely male, less well educated, less likely to be married/in a de facto relationship, and more likely to have ever smoked. In addition, they were taller with a lower BMI but higher ABSI, were older at diabetes diagnosis but had a longer duration of diabetes, and were more likely insulin treated. They had a lower serum total cholesterol but were more often statin, if not fibrate, treated. They were more likely to have chronic renal impairment, a raised urinary albumin:creatinine ratio (uACR), CVD, and a history of heart failure.

**Table 1.** Baseline characteristics of pooled participants with type 2 diabetes from the Fremantle Diabetes Study Phases I and II categorized by prevalent distal symmetric polyneuropathy status assessed from a 6-point or 2-point Michigan Neuropathy Screening Instrument score

		6-point MNSI			2-point MNSI	
	No prevalent DSPN	Prevalent DSPN	*P*	No prevalent DSPN	Prevalent DSPN	*P*
No. (%)	1717 (63.5)	987 (36.5)		1715 (63.6)	981 (36.4)	
FDS phase II, %	55.0	53.0	.336	53.6	58.7	.011
Age, y	61.9 ± 11.2	69.8 ± 10.2	<.001	61.6 ± 11.3	70.3 ± 9.5	<.001
Male, %	47.2	56.6	<.001	44.1	61.5	<.001
Ethnic background, %			<.001			<.001
Anglo-Celt	53.6	61.0		54.0	60.3	
Southern European	16.4	13.5		16.7	12.6	
Other European	7.7	8.7		7.1	9.2	
Asian	4.9	2.4		4.5	3.2	
Aboriginal	4.1	4.0		4.7	3.4	
Mixed/other	13.2	10.4		12.9	11.3	
Not fluent in English, %	12.9	13.4	.722	12.5	13.6	.437
Educated beyond primary school level, %	82.6	77.7	.002	83.1	78.0	.001
Married/de facto relationship, %	67.4	59.3	<.001	66.2	61.0	.007
Smoking status, %			.042			<.001
Never	46.3	42.8		47.2	41.2	
Ex-	40.8	45.8		39.3	48.2	
Current	12.8	11.4		13.5	10.6	
Alcohol consumption, standard drinks/d	0.1 (0-0.8)	0.1 (0-0.8)	.286	0.1 (0-0.8)	0.1 (0-1.2)	.510
Height, cm	165.0 ± 9.6	167.0 ± 10.0	<.001	164.4 ± 9.5	167.8 ± 10.0	<.001
Body mass index	30.6 ± 6.0	30.2 ± 5.6	.061	30.8 ± 6.1	30.0 ± 5.5	<.001
A body shape index (m^11/6^/kg^2/3^)	0.081 ± 0.005	0.083 ± 0.005	<.001	0.081 ± 0.005	0.083 ± 0.005	<.001
Central adiposity, %*^a^*	66.7	69.7	.113	67.6	68.2	.764
Age at diagnosis, y	55.0 ± 11.7	59.5 ± 12.1	<.001	54.7 ± 11.8	60.1 ± 11.8	<.001
Diabetes duration, y	4.4 (1.0-11.0)	8.0 (3.0-15.8)	<.001	4.1 (1.0-11.0)	8.0 (3.0-16.0)	<.001
Fasting serum glucose, mmol/L	7.5 (6.2-9.4)	7.6 (6.3-10.0)	.046	7.6 (6.2-9.6)	7.5 (6.2-9.5)	.402
HbA_1c_, %	6.9 (6.2-8.1)	7.1 (6.4-8.3)	<.001	7.0 (6.2-8.1)	7.0 (6.2-8.1)	.751
Diabetes treatment, %			<.001			<.001
Diet	32.3	21.1		31.4	23.0	
Oral agents/noninsulin injectables	53.5	56.6		54.7	54.7	
Insulin ± oral agents/injectables	14.3	22.3		13.9	22.2	
Systolic blood pressure, mm Hg	145 ± 22	152 ± 24	<.001	145 ± 22	152 ± 24	<.001
Diastolic blood pressure, mm Hg	80 ± 11	80 ± 12	.629	80 ± 12	80 ± 12	.871
Blood pressure–lowering medication, %	60.1	67.5	<.001	59.3	69.4	<.001
Heart rate, bpm	69 ± 12	70 ± 13	.022	69 ± 12	70 ± 13	.165
Total serum cholesterol, mmol/L	4.9 ± 1.2	4.8 ± 1.2	.032	4.9 ± 1.2	4.7 ± 1.2	<.001
Serum HDL cholesterol, mmol/L	1.15 ± 0.34	1.16 ± 0.34	.737	1.15 ± 0.34	1.16 ± 0.35	.378
Serum total:HDL cholesterol ratio	4.3 (3.0-6.2)	4.2 (2.9-6.1)	.075	4.3 (3.0-6.4)	4.1 (2.8-5.9)	<.001
Serum non-HDL cholesterol, mmol/L	3.7 ± 1.2	3.6 ± 1.2	.035	3.8 ± 1.2	3.6 ± 1.2	<.001
Serum triglycerides, mmol/L	1.8 (1.0-3.3)	1.8 (1.0-3.0)	.501	1.8 (1.0-3.3)	1.7 (1.0-3.0)	.016
Lipid-lowering medication, %						
Any	42.4	41.3	.599	40.8	46.1	.010
Statins	39.1	39.3	.935	37.7	43.6	.003
Fibrates	3.4	1.8	.021	3.4	1.8	.016
Aspirin use, %	29.4	31.4	.258	27.3	36.1	<.001
eGFR category, mL/min/1.73 m^2^, %			<.001			<.001
≥90	43.9	22.5		44.2	22.1	
60-89	44.0	52.3		44.2	52.4	
45-59	8.0	13.6		7.7	13.6	
<45	4.1	11.6		3.9	11.8	
Urinary albumin:creatinine, mg/mmol	3.4 (1.0-11.1)	5.4 (1.2-23.1)	<.001	3.4 (1.0-11.5)	5.1 (1.2-21.1)	<.001
Coronary heart disease, %	24.6	35.9	<.001	24.8	35.4	<.001
Heart failure, %	5.0	10.7	<.001	5.1	10.3	<.001
Cerebrovascular disease, %	7.7	14.9	<.001	7.5	15.8	<.001
Peripheral arterial disease, %	23.2	29.0	.001	22.2	30.0	<.001

Abbreviations: bpm, beats per minute; DSPN, distal symmetric polyneuropathy; eGFR, estimated glomerular filtration rate; FDS, Fremantle Diabetes Study; HbA_1c_, glycated hemoglobin A_1c_; HDL, high-density lipoprotein; MNSI, Michigan Neuropathy Screening Instrument.

^*a*^Waist circumference 94.0 cm or greater in men, 80.0 cm or greater in women.

Logistic regression models of the independent associates of prevalent DSPN are summarized in Table 2. The significant coincident risk factors for DSPN detected by 6- and 2-point MNSI were older age, Aboriginal ethnic background, greater height, diabetes duration, insulin treatment, and higher uACR. Additional risk factors for 6-point MNSI DSPN were marital status (protective), greater central obesity by waist circumference, a higher glycated hemoglobin A_1c_ (HbA_1c_), noninsulin blood glucose-lowering therapy, and eGFR less than 30 mL/min/1.73m^2^, while for 2-point MNSI DSPN they were Southern European ethnicity (protective), education beyond primary level (protective), greater BMI, and greater ABSI. When FDS phase was added to the most parsimonious models, participants in FDS2 had a 32% lower risk of DSPN vs those in FDS1 for 6-point MNSI but this variable was not significant for 2-point MNSI. Introduction of FDS phase rendered eGFR greater than 90 mL/min/1.73m^2^ nonsignificant in the 6-point MNSI logistic regression model.

**Table 2.** The most parsimonious multiple logistic regression model of independent associates of prevalent distal symmetric polyneuropathy assessed from a 6-point and 2-point Michigan Neuropathy Screening Instrument scores in pooled participants with type 2 diabetes from the Fremantle Diabetes Study (FDS) phases I and II with FDS phase entered afterward

	6-point MNSI		2-point MNSI	
	OR (95% CI)	*P*	OR (95% CI)	*P*
Age, increase of 1 y	1.08 (1.07-1.09)	<.001	1.10 (1.08-1.11)	<.001
Aboriginal ethnicity	2.31 (1.43-3.73)	<.001	1.82 (1.09-3.02)	.021
Southern European ethnicity			0.68 (0.50-0.92)	.012
Married/de facto relationship	0.80 (0.66-0.96)	.017		
Education beyond primary level			0.75 (0.57-0.98)	.032
Height, increase of 1 cm	1.05 (1.04-1.06)	<.001	1.07 (1.06-1.08)	<.001
Central adiposity*^a^*	1.36 (1.12-1.64)	.002		
ABSI, increase of 0.001 m^11/6^/kg^2/3^			1.03 (1.01-1.05)	.005
Body mass index, increase of 1			1.02 (1.00-1.04)	.014
Diabetes duration, increase of 1 y	1.02 (1.01-1.03)	.003	1.02 (1.01-1.04)	.002
HbA_1c_, increase of 1% or 11 mmol/mol	1.09 (1.02-1.16)	.007		
On oral agents/noninsulin injectables	1.33 (1.06-1.66)	.013		
On insulin ± other agents	1.58 (1.15-2.17)	.005	1.43 (1.10-1.85)	.007
Ln(uACR), mg/mmol*^b^*	1.11 (1.03-1.19)	.006	1.16 (1.08-1.24)	<.001
eGFR >90 mL/min/1.73m^2^	0.84 (0.67-1.04)	.112		
eGFR <30 mL/min/1.73m^2^	2.02 (1.05-3.90)	.036		
FDS2 vs FDS1	0.68 (0.56-0.83)	<.001	0.90 (0.74-1.11)	.326

FDS phase replaced one previously significant variable. Data are ORs and 95% CIs.

Abbreviations: ABSI, A Body Shape Index; eGFR, estimated glomerular filtration rate; FDS1, Fremantle Diabetes Study phase 1; FDS2, Fremantle Diabetes Study phase 2; HbA_1c_, glycated hemoglobin A_1c_; MNSI, Michigan Neuropathy Screening Instrument; OR, odds ratio; uACR, urinary albumin:creatinine ratio.

^*a*^Waist circumference 88.0 cm or greater in women, 102.0 cm or greater in men.

^*b*^uACR – an increase of 1 in ln(uACR) equates to an increase of 2.72 in uACR.

### Incident Distal Symmetric Polyneuropathy

For DSPN detected by 6-point MNSI, there were 245 FDS1 participants who developed DSPN by year 4 during 1828 person-years (3.9 ± 1.1 years) of follow-up (see Fig. 1), an IR and 95% CI of 13.6 (12.0-15.4)/100 person-years, equivalent to 13.6%/year. In FDS2, 390 developed DSPN by year 4 during 2218 person-years (3.3 ± 1.2 years; see Fig. 2), an IR of 17.6 (15.9-19.4)/100 person-years, equivalent to 17.6%/year. The IRR of FDS2 vs FDS1 was 1.31 (1.12-1.55) (*P* = .001) and the IRD 4.18 (1.76-6.60)/100 person-years (*P* = .001).

For DSPN detected by 2-point MNSI, there were 252 FDS1 participants who developed DSPN by year 4 during 1809 person-years (3.8 ± 1.2 years) of follow-up (see Fig. 1), an IR of 13.9 (12.3-15.8)/100 person-years, equivalent to 13.9%/year. In FDS2, 177 developed DSPN by year 4 during 2399 person-years (3.8 ± 1.0 years; see Fig. 2), an IR of 7.4 (16.3-8.6)/100 person-years, equivalent to 7.4%/year. The IRR of FDS2 vs FDS1 was 0.53 (0.43-0.64) (*P* < .001) and the IRD −6.55 (−8.58 to −4.51)/100 person-years (*P* < .001).

The characteristics of pooled participants in FDS1 and FDS2 by incident DSPN status assessed by 6-point or 2-point MNSI score are shown in Table 3. There were fewer significant shared bivariable associations relative to prevalent DSPN. Compared to those without incident DSPN, participants with incident DSPN were older at baseline and diabetes diagnosis, had a higher HbA_1c_ and systolic blood pressure, and were more likely to have chronic renal impairment and CHD.

**Table 3.** Baseline characteristics of pooled participants with type 2 diabetes from the Fremantle Diabetes Study phases I and II categorized by incident distal symmetric polyneuropathy status over 4 years assessed from a 6-point or 2-point Michigan Neuropathy Screening Instrument score

		6-point MNSI			Two-point MNSI	
	No incident DSPN	Incident DSPN	*P*	No incident DSPN	Incident DSPN	*P*
No., %	516 (44.8)	635 (55.2)		680 (61.3)	429 (38.7)	
FDS phase II, %	55.6	61.4	.047	67.5	41.3	<.001
Age, y	60.0 ± 9.9	63.3 ± 10.6	<.001	59.7 ± 10.2	65.3 ± 9.8	<.001
Male, %	49.6	50.1	.906	44.4	52.7	.008
Ethnic background, %			.837			<.001
Anglo-Celt	56.4	57.8		56.2	62.0	
Southern European	14.1	15.4		13.8	17.5	
Other European	7.9	6.9		6.0	8.4	
Asian	5.6	4.3		5.7	1.4	
Aboriginal	2.9	2.5		2.8	1.9	
Mixed/other	13.0	13.1		15.4	8.9	
Not fluent in English, %	9.9	10.9	.628	8.7	13.5	.012
Educated beyond primary level, %	86.9	83.8	.154	88.3	80.9	<.001
Married/de facto relationship, %	70.2	68.3	.522	70.7	65.7	.084
Smoking status, %			.811			.105
Never	49.1	47.2		50.9	44.4	
Ex-	40.8	42.0		39.1	44.6	
Current	10.1	10.7		10.0	11.0	
Alcohol use, standard drinks/d	0.1 (0-0.8)	0.1 (0-1.2)	.254	0.1 (0-0.8)	0.1 (0-1.2)	.828
Height, cm	165.6 ± 9.0	165.9 ± 9.8	.540	164.8 ± 9.2	166.2 ± 9.7	.020
Body mass index	30.8 ± 6.0	30.2 ± 5.4	.083	30.9 ± 5.9	30.5 ± 5.8	.207
A Body Shape Index (m^11/6^/kg^2/3^)	0.080 ± 0.005	0.081 ± 0.005	.053	0.080 ± 0.005	0.081 ± 0.005	<.001
Central adiposity, %*^a^*	66.4	65.5	.755	66.4	68.2	.555
Age at diagnosis, y	54.1 ± 10.6	56.0 ± 11.4	.005	53.4 ± 10.6	58.2 ± 10.6	<.001
Diabetes duration, y	3.1 (1.0-9.0)	5.0 (1.1-12.0)	<.001	4.0 (1.0-10.0)	4.6 (1.5-11.0)	.059
Fasting serum glucose, mmol/L	7.2 (6.2-9.1)	7.5 (6.3-9.2)	.240	7.2 (6.2-8.8)	7.6 (6.3-10.0)	.002
HbA_1c_,%	6.8 (6.0-7.8)	6.9 (6.2-8.1)	.015	6.8 (6.1-7.7)	7.0 (6.2-8.2)	.003
Diabetes treatment, %			.009			.077
Diet	35.7	29.3		35.2	29.1	
Oral agents/noninsulin injectables	52.6	53.6		51.6	54.5	
Insulin ± oral agents/injectables	11.7	17.1		13.3	16.4	
Systolic blood pressure, mm Hg	142 ± 19	146 ± 22	<.001	142 ± 19	149 ± 21	<.001
Diastolic blood pressure, mm Hg	80 ± 11	81 ± 12	.593	81 ± 11	81 ± 12	.972
Blood pressure–lowering medication, %	56.9	65.2	.004	59.9	61.3	.659
Heart rate, bpm	68 ± 10	69 ± 12	.022	69 ± 11	69 ± 13	.908
Total serum cholesterol, mmol/L	4.9 ± 1.2	4.8 ± 1.3	.441	4.8 ± 1.2	5.0 ± 1.2	.101
Serum HDL-cholesterol, mmol/L	1.14 ± 0.32	1.17 ± 0.34	.181	1.18 ± 0.32	1.14 ± 0.36	.032
Serum total:HDL cholesterol ratio	4.3 (3.0-6.2)	4.2 (2.9-6.1)	.141	4.1 (2.9-5.8)	4.4 (3.0-6.7)	<.001
Serum non-HDL cholesterol, mmol/L	3.7 ± 1.2	3.7 ± 1.2	.252	3.6 ± 1.2	3.8 ± 1.2	.003
Serum triglycerides, mmol/L	1.8 (1.0-3.1)	1.8 (1.0-3.3)	.932	1.7 (1.0-2.9)	1.9 (1.0-3.5)	.007
Lipid-lowering medication, %						
Any	44.1	46.9	.341	48.7	37.3	<.001
Statins	39.8	44.1	.150	45.7	33.3	<.001
Fibrates	3.9	3.3	.634	3.4	4.4	.421
Aspirin use, %	28.2	32.5	.122	26.4	30.5	.149
eGFR category, mL/min/1.73 m^2^, %			.015			<.001
≥90	48.5	42.2		49.9	34.5	
60-89	43.3	46.6		44.1	49.8	
45-59	6.2	6.3		4.6	10.3	
<45	1.9	4.9		1.5	5.4	
Urinary albumin:creatinine, mg/mmol	2.7 (1.0-7.6)	3.5 (1.0-11.5)	<.001	2.6 (0.9-7.5)	3.8 (1.2-12.6)	<.001
Coronary heart disease, %	19.6	26.5	.006	18.2	30.5	<.001
Heart failure, %	2.7	4.7	.089	1.9	5.6	.002
Cerebrovascular disease, %	5.6	8.2	.105	5.9	7.9	.216
Peripheral arterial disease, %	20.0	22.0	.425	18.6	22.9	.091

Abbreviations: bpm, beats per minute; DSPN, distal symmetric polyneuropathy; eGFR, estimated glomerular filtration rate; FDS, Fremantle Diabetes Study; HbA_1c_, glycated hemoglobin A_1c_; HDL, high-density lipoprotein; MNSI, Michigan Neuropathy Screening Instrument.

^*a*^Waist circumference 94.0 cm or greater in men, 80.0 cm or greater in women.

Weibull interval-censored regression models of the independent determinants of incident DSPN are summarized in Table 4. There was 1 significant coincident risk factor for DSPN detected by 6- and 2-point MNSI, namely increasing age. Additional risk factors for 6-point MNSI DSPN were a systolic blood pressure and insulin therapy, while for 2-point MNSI DSPN they were Asian ethnicity (inversely), lack of English fluency, greater height, a higher fasting serum glucose and uACR, and eGFR less than 30 mL/min/1.73m^2^. Participants in FDS2 had a 25% greater risk of incident 6-point MNSI DSPN vs those in FDS1 after adjustment but, for 2-point MNSI DSPN, FDS2 was associated with a more than halving of incident DSPN risk. Introduction of this variable into the most parsimonious models had no effect on independent variables for 6-point MNSI DSPN but rendered current smoking, statin therapy, and CHD nonsignificant in the case of 2-point MNSI DSPN.

**Table 4.** The most parsimonious interval-censored Weibull regression model of independent baseline determinants of incident distal symmetric polyneuropathy by the year 4 assessment in participants with type 2 diabetes from the Fremantle Diabetes Study (FDS) phases I and II with FDS phase entered afterward

	6-point MNSI		2-point MNSI	
	HR (95% CI)	*P*	HR (95% CI)	*P*
Age, increase of 1 y	1.02 (1.01-1.03)	<.001	1.07 (1.05-1.08)	<.001
Asian ethnicity			0.38 (0.17-0.86)	.021
Not fluent in English			1.36 (1.02-1.82)	.039
Current smoker			1.27 (0.93-1.74)	.140
Height, increase of 1 cm			1.04 (1.03-1.05)	<.001
Systolic blood pressure, increase of 10 mm Hg	1.06 (1.02-1.10)	.004		
Fasting serum glucose, increase of 1 mmol/L			1.04 (1.01-1.08)	.019
Insulin therapy	1.29 (1.04-1.60)	.022		
Statin therapy			0.97 (0.74-1.28)	.846
Coronary heart disease			1.23 (0.97-1.56)	.087
Ln(uACR), mg/mmol*^a^*			1.12 (1.03-1.22)	.011
eGFR <30 mL/min/1.73 m^2^			3.55 (1.12-11.19)	.031
FDS2 vs FDS1	1.25 (1.06-1.48)	.009	0.42 (0.33-0.55)	<.001

FDS phase replaced some previously significant variables. Data are HRs and 95% CIs.

Abbreviations: ABSI, A Body Shape Index; eGFR, estimated glomerular filtration rate; FDS1, Fremantle Diabetes Study phase 1; FDS2, Fremantle Diabetes Study phase 2; HbA_1c_, glycated hemoglobin A_1c_; HR, hazard ratio; MNSI, Michigan Neuropathy Screening Instrument; OR, odds ratio; uACR, urinary albumin:creatinine ratio.

^*a*^uACR – an increase of 1 in ln(uACR) equates to an increase of 2.72 in uACR.

## Discussion

The present data are the first to examine temporal changes in DSPN complicating type 2 diabetes in cohorts of representative, community-based participants. They show that the prevalence of DSPN in FDS2 was less than or equivalent to that in FDS1 after adjustment for confounding variables depending on whether a 6- or 2-point MNSI score was used to ascertain DSPN. For incident DSPN over 4 years in adjusted statistical models, there was a mildly increased risk using a 6-point MNSI but a significantly lower risk (an approximate halving) for 2-point MNSI based on biothesiometry alone. Other associates/predictors of DSPN varied between the methods of ascertainment apart from increasing age, which was present in all prevalent and incident analyses. Based on the more robust 2-point vs 6-point MNSI score in multivariable models adjusting for confounders, these data suggest that the prevalence of DSPN had not changed between FDS1 and FDS2, but that the subsequent incidence was lower in FDS2 consistent with the reductions in DSPN incidence between 1996 and 2018, as ascertained from VPT alone in the Danish tertiary center study (19). The differences between 8-point, 6-point, and 2-point MNSI in the present study raise questions concerning the methods used for bedside assessment of this complication in type 2 diabetes, with implications for within- and between-study DSPN comparisons.

Although estimates of the prevalence of DSPN in epidemiologic studies vary widely, the high rate in FDS2 as ascertained from the full 8-point MNSI (close to 60%) is greater than the range found in other studies (up to 50% (44, 45)) as well as in FDS1, and in both phases using 6- and 2-point MNSI scores (<39%). This suggests that foot abnormalities on inspection had either increased between FDS1 and FDS2 or, given that this is the most subjective part of the assessment, that they were more readily detected and reported in FDS2. The latter explanation appears more likely given that other aspects of the MNSI score had not changed as much between phases and, as acknowledged earlier, probably reflected the combination of high general population background rates of skin abnormalities including callus/corns (28, 29) and especially xerosis cutis (27), but also deformities such as flat feet and hallux valgus (28), coupled with greater awareness by FDS2 participants and staff. Further assessment of this hypothesis was beyond the scope of the present study.

There were also differences in our findings when a 6-point vs 2-point MNSI was used in analyses. Given the low rate of foot ulceration in our participants (36), these differences primarily reflect the effect of adding ankle reflexes to VPT in MNSI grading. These 2 assessment modalities assess large fiber neuropathy, are affected by increasing age (37–39), and have comparable and acceptable interrater reliability (42). VPT, but not ankle reflexes, has been validated against NCS (41). There are few data that have allowed a direct comparison between them in the context of DSPN diagnosis in type 2 diabetes. However, one study found that ankle reflexes had moderate diagnostic accuracy (77.0%), 64.7% sensitivity, and 80.3% specificity compared with biothesiometry (46). In addition, while there was significant overlap in the significant associates of prevalent DSPN using 6- and 2-point MNSI (see Table 2), including the well-recognized risk factors diabetes duration (47), obesity (47), height (48), and microalbuminuria (49), it is possible that renal impairment (50) and poor glycemic control (51) were significant for 6-point rather than 2-point MNSI due to their association with xerosis rather than nerve fiber dysfunction. Furthermore, VPT is a significant predictor both of foot ulceration (52) and death (53). Given this range of considerations, we would put more weight on the 2-point compared with 6-point MNSI DSPN prevalence data.

Reports of annual IRs for DSPN complicating type 2 diabetes have varied from 2% to 8% (54, 55), reflecting heterogeneity in participant sample characteristics and methods of DSPN ascertainment. The 2-point MNSI DSPN IRs in FDS1 (13.9%/year) and FDS2 (7.4%/year) were relatively high. This probably reflects the fact that our participants were older and had longer duration disease than most cohorts with lower IRs (55), but they were lower than those in heavily selected participants in the Danish tertiary center study (19). The IR in FDS2 was approximately half that in FDS1 and, in the incidence regression model incorporating the 2-point MNSI score, the hazard ratio for FDS2 vs FDS1 was of similar magnitude. These findings suggest that the lower incidence of DSPN in FDS2 was independent of beneficial temporal changes in risk factors such as glycemic control (47). We assume that the multivariable model did not capture the effect of important between-phase differences in key risk factors and their potential interactions, or there were unknown risk factors. Nevertheless, the lower risk of incident DSPN in FDS2 vs FDS1 independent of recognized risk factors paralleled that for CVD, heart failure, LEA, and CVD-related death in our cohorts (5).

There were sociodemographic variables that were influential in our models of prevalent and incident DSPN. Although Australian Aboriginal ethnicity was positively associated with prevalent DSPN, as previously reported (56), Southern European and Asian ethnicities were associated with a lower risk of prevalent and incident DSPN, respectively, as assessed from the 2-point MNSI. A lack of fluency in English, which was most frequent among Southern European participants in the FDS, was also associated with a lower incidence of DSPN ascertained from the 2-point MNSI. It is possible that the relatively low DSPN rate in our Southern European participants reflects their Mediterranean diet and its beneficial effects on oxidant stress (57). A number of studies have shown that White ethnicity is associated with a higher risk of DSPN than in Asian individuals in the absence of a clear explanation (58). It has been hypothesized that lower rates of smoking and hypertriglyceridemia may underlie the reduced susceptibility to DSPN in Asians (59). Smoking, but not serum triglycerides, was a significant predictor of incident DSPN in the present study. However the rate of current smoking among Asians did not differ significantly from other ethnic groups in FDS2, while there was no significant interaction between Asian ethnicity and age for incident DSPN even though the Asians were younger than other racial subgroups (*P* ≥ .798 in both cases).

The present study had limitations. There may have been selection bias in both FDS phases in that despite age, sex, and proportions by diabetes type of participants and nonparticipants in FDS1 and FDS2 being similar (20), healthier residents with diabetes may have participated. In addition, the subgroups of FDS1 and FDS2 participants with serial MNSI scores may have represented a selected sample given that approximately one-third of eligible participants were not included in incidence analyses because of missing data. The research nurses who collected MNSI data changed between and within FDS phases but all were trained in the requisite clinical techniques, notwithstanding that the assessment of foot appearance is subjective. As is being implemented for assessment of foot ulcers (60), future studies should attempt to develop rigorous abnormal appearance criteria with multiperson adjudication. A major strength of the present study is that the FDS is one of the largest and longest-running diabetes-specific natural history studies yet conducted, involving well-characterized participants.

The acknowledged reference diagnostic test for DSPN is NCS but, for reasons of cost, convenience, patient comfort, and availability, it is not a routine clinical investigation and has its own limitations (61). There is no universally endorsed alternative (44). Our data support use of the VPT as a simple, relatively robust bedside assessment of DSPN complicating diabetes that has been used in intervention trials (40) and that requires relatively inexpensive equipment and is easy to use. The inclusion of other aspects of the MNSI is questionable, especially foot appearance, which may benefit from the development of more objective assessment criteria.

In conclusion, we would recommend VPT as the most objective and robust measure for incorporation in epidemiologic studies, notwithstanding the fact that multifaceted assessment through the MNSI generates useful information on other aspects of foot health that have clinical management implications. The present detailed longitudinal study of community-based people with type 2 diabetes has shown that, although the prevalence of DSPN remained stable during the 15 years between FDS phases, its incidence has reduced after adjustment for explanatory and confounding variables as assessed using VPT alone. Other independent predictors of incident DSPN in our participants, including glycemic control, microalbuminuria, and renal impairment, suggest a range of management strategies that could help prevent the development of this potentially debilitating complication.

## Data Availability

Restrictions apply to the availability of data generated or analyzed during this study to preserve patient confidentiality or because they were used under license. The corresponding author will on request detail the restrictions and any conditions under which access to some data may be provided.

## Abbreviations

ABSI: A Body Shape Index
BMI: body mass index
CHD: coronary heart disease
CVD: cardiovascular disease
DSPN: distal symmetric polyneuropathy
eGFR: estimated glomerular filtration rate
FDS: Fremantle Diabetes Study
HbA_1c_: glycated hemoglobin A_1c_
HDL: high-density lipoprotein
IR: incidence rate
IRD: incident rate difference
IRR: incidence rate ratio
LEA: lower extremity amputation
MNSI: Michigan Neuropathy Screening Instrument
NCS: nerve conduction studies
uACR: urinary albumin:creatinine ratio
VPT: vibration perception threshold
